# A Novel Method of Treatment for a Mal-United Galeazzi Fracture With Dislocation of the Distal Radioulnar Joint Using Scarf Osteotomy

**DOI:** 10.7759/cureus.14276

**Published:** 2021-04-03

**Authors:** Yasmeen Khan, George Hourston, Phillip Johnston

**Affiliations:** 1 Department of Trauma and Orthopaedics, Addenbrooke's Hospital, Cambridge University Hospitals NHS Foundation Trust, Cambridge, GBR

**Keywords:** scarf osteotomy, distal radio-ulnar joint, galeazzi fracture

## Abstract

The Galeazzi fracture is an unstable fracture-dislocation of the forearm. There have been reports of non-union of the radius despite rigid internal fixation with a plate. We present the case of a 25-year-old male who fell from his bicycle, sustaining a closed Galeazzi fracture-dislocation. Definitive surgical fixation involved internal fixation using a six-hole dynamic compression plate. Post-operatively, the patient noted a significant reduction in pronation. The fracture had united but with approximately 5 mm of radial shortening. The operating consultant formulated a surgical plan to resolve the complex nature of this mal-united Galeazzi fracture. A scarf-type osteotomy would correct the deformity and reduce the risk of non-union. Clinically and radiographically, the scarf osteotomy had healed by three months. The patient was very pleased that he underwent revision surgery, as the pain from the wrist resolved and the range of movement improved.

## Introduction

The Galeazzi fracture is an unstable fracture-dislocation of the forearm. Misdiagnosis or inadequate management of a Galeazzi fracture may result in disabling complications. There have been reports of non-union of the radius despite rigid internal fixation with a plate [1]. We describe here a novel surgical method for the treatment of a chronic mal-united Galeazzi fracture with dislocated distal radioulnar joint (DRUJ) using scarf-type osteotomy of the distal radius.

## Case presentation

A 25-year-old mechanical robotic engineer fell from his bicycle at 25 mph, landing on to his left non-dominant forearm. He had no significant past medical or surgical history. He sustained a closed left Galeazzi fracture-dislocation with a dorsally displaced ulna head. Following initial assessment in Accident and Emergency, he underwent manipulation under sedation and application of an above-elbow back slab for comfort. Definitive surgical fixation involved a volar approach to the forearm, open reduction and internal fixation using a six-hole low contact dynamic compression plate. The surgical team found difficulty in reducing the DRUJ, although it was left in a satisfactorily reduced position. The forearm was immobilised in an above-elbow cast in supination.

The patient attended regular follow-up clinics and six weeks post-operatively, he noted a significant reduction in pronation. Nine months following his initial injury, he was referred to the hand surgical team for a second opinion of his left wrist following the Galeazzi fracture-dislocation non-union. This was treated with open reduction and internal fixation; no bone grafting was performed. A straight plate was applied in compression mode. The post-reduction radiographs showed a subtle flexion deformity of the radial shaft. The DRUJ remained congruent and reduced on radiographs. The fracture had united but with approximately 5 mm of radial shortening.

On examination, he had crepitus of the radial-sided tendons in the forearm as they ran over the tips of the screws in the radial shaft. The radius had a subtle flexion bow and reduced rotation. The range of motion was limited to 45 degrees supination and 50 degrees pronation. The wound had healed, and there was no distal neurological deficit.

The senior author formulated a surgical plan to resolve the complex nature of this mal-united Galeazzi fracture. A distal radius osteotomy would correct both the shortening of the radius and the palmar angulation. The simplest would be an opening wedge but would result in a risk of non-union [2]. A scarf-type osteotomy, which is ‘z-shaped’ in the anteroposterior view, would be more complex but would reduce this risk of non-union. This step-cut lengthening z osteotomy could be performed without bone grafting. Plate replacement would also be necessary to support the osteotomy.

**Figure 1:**
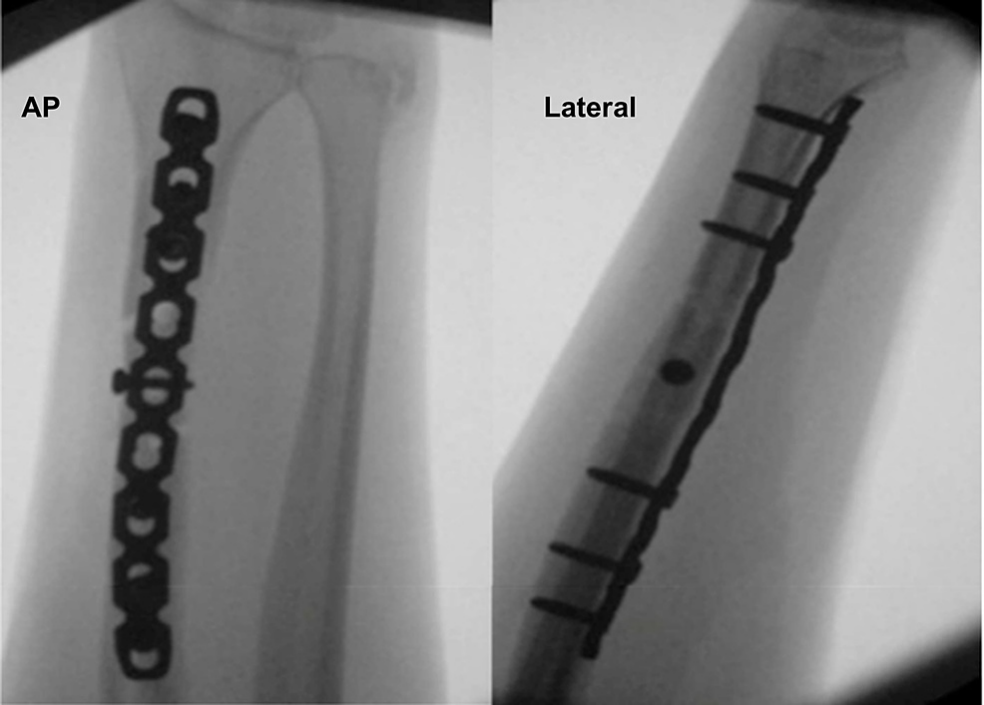
Intra-operative fluoroscopic images (antero-posterior and lateral views) showing scarf osteotomy

Fourteen months following the initial injury, he underwent revision surgery on his left forearm. Under general anaesthetic with a regional block and the arm tourniquet inflated to 250 mmHg for 57 minutes, a corrective scarf type distal radius osteotomy was performed. The procedure involved Henry’s approach to the distal half of the volar forearm reopening the previous interval with proximal extension for re-fixation. The radial artery left within a fascial scar envelope was retracted radially. The flexor carpi radialis, flexor pollicis longus and pronator teres were taken ulnar-wards. Pronator quadratus was incised on its radial border to expose the distal radius. The old metalwork was removed; the plate was found to have been contoured into flexion for the previous fixation. A scarf-osteotomy was performed via holes three-five of the previous fixation, with continuous hand cooling (saline) and dorsal soft tissue protection. A nine-hole locking compression reconstruction (LCP recon) plate contoured for radial bow and to allow a little (approximately 10°) extension at the osteotomy site was applied distally with good hold. No bone grafting was used. The length of the construct was adjusted according to the positive ulnar variance noted on the pre-operative wrist radiograph. The plate was secured proximally; the osteotomy then was compressed and an inter-fragmentary compression screw placed with excellent hold (Figure 1). The DRUJ was stable post-fixation. Manipulation under anaesthetic revealed 60° of supination and 90° pronation.

The patient’s postoperative course was uneventful. He engaged with gentle active motion after two weeks. Clinically and radiographically, the scarf osteotomy had healed by the three months' follow-up. On examination, he had supination of 50°, pronation of 70°, full wrist extension but flexion to 50°. He was very pleased that he underwent revision surgery, as the pain from the wrist and the range of movement improved.

## Discussion

Galeazzi fracture malunion is an uncommon occurrence and its management is challenging. To our knowledge, there have been no reports on the use of a scarf-type radial correction osteotomy for the surgical treatment of a mal-united Galeazzi fracture with a chronic DRUJ. Wedge osteotomies are more commonly used for the reconstruction of malunited diaphyseal forearm fractures [3]. These can correct axial, rotational, and angular deformities. While wedge osteotomies can achieve lengthening, bone grafting may be required to achieve this [3]. Z-shaped scarf osteotomies have been more commonly employed for metatarsal hallux valgus corrective surgery [4]. They are considered stable and versatile.

A Galeazzi fracture results in a complex traumatic disruption of the DRUJ that is associated with an unstable fracture, commonly at the junction of the middle and distal third of the radial shaft [5-6]. They comprise about 3% of all fractures of the forearm [7]. The typical mechanism of injury is a fall with forceful axial loading and torsion of the forearm with the wrist hyperextended and pronated [1]. They are usually treated surgically, with good to excellent functional results in 80% to 95% of patients [8-10].

Diagnosis is established on radiographic evaluation. Nonsurgical management with anatomic reduction and immobilization in a long-arm cast has been successful in children [10-11]. In adults, Galeazzi fractures are extremely unstable; nonsurgical treatment typically fails because of deforming forces of the pronator quadratus, brachioradialis, thumb abductors and extensors muscles. Standard surgical management comprising open reduction and internal fixation of the radial shaft fracture is well-described in literature [5-6,11-13]. Disruption of the ligamentous complex, including the DRUJ, triangular fibrocartilage complex and the interosseous membrane are commonly overlooked. Anatomic reduction and stable rigid fixation of the radius should be followed by intraoperative assessment of the DRUJ [10-11].

The most devastating complication of this fracture type is mal-union of the radius with chronic subluxation and instability of the DRUJ. Patients may experience persistent pain, limited forearm rotation, and loss of grip strength. Corrective osteotomy is the main treatment for this complication [3]. The patient described in this report underwent a z-shaped scarf type radial correction osteotomy to achieve lengthening and improve DRUJ stability without the requirement for bone grafting.

## Conclusions

Galeazzi fracture non-union is uncommon, and its management is challenging. We have successfully treated this complicated fracture, which results in significant morbidity if neglected, with a radial shaft scarf osteotomy for acquired shortening and flexion deformity after fixation of the Galeazzi fracture-dislocation.
